# Factors influencing healthcare providers’ attitude and willingness to use information technology in diabetes management

**DOI:** 10.1186/s12911-021-01398-w

**Published:** 2021-01-21

**Authors:** Binyam Tariku Seboka, Tesfahun Melese Yilma, Abraham Yeneneh Birhanu

**Affiliations:** 1 Department of Health Informatics, School of Public Health, Dilla University, Dilla, Ethiopia; 2 Department of Health Informatics, Institute of Public Health, University of Gondar, Gondar, Ethiopia

**Keywords:** Information technology, Remote monitoring, Healthcare providers, Diabetes, Attitude, Willingness, Ethiopia

## Abstract

**Background:**

The use of information technologies could help to improve communications between patients and care providers, might improve overall patient management practice. However, the potential for implementing these patient management options in Ethiopia has not been well documented. This institution-based survey aimed to describe the attitude and willingness of care providers towards the use of information technologies for managing diabetes patients, and factors influencing their interest.

**Methods:**

A cross-sectional quantitative survey was conducted on 423 study participants from February to March 2020 at two teaching hospitals in Northwest Ethiopia, where remote monitoring patients had not been implemented. A pretested self-administered questionnaire was used to collect the required data. Other than descriptive statistics, the binary logistic regression analysis method was used to identify factors associated with attitude. Also, the negative binomial regression method was used to identify factors associated with willingness to use information technologies.

**Result:**

A total of 406 participants (69.7%, n = 283 nurses and 30.3%, n = 123 physicians) were completed survey. Overall, 64% of respondents had a favorable attitude towards remote monitoring, and the majority of them were willing to use voice call (74.4%), text message (62.1%), video conference (61.3%), e-mail (60.6%), and social media (57.4%) as a source of communication to support patients. The result of regression analysis shows that having a computer (AOR = 2.3, 95% CI: [1.3, 3.8]), innovativeness (AOR = 2.8, 95% CI: [1.8, 4.3]), and practice of supporting patient by electronic technologies (AOR = 1.7, 95% CI: [1.1, 2.6]) were significantly associated with attitude to remote monitoring. Attitude towards remote monitoring (IRR = 2.3, 95% CI: 1.1–4.7), computer use (IRR = 1.3, 95% CI: 1.162–3.023), frequently searching health-related information (IRR = 1.7, 95% CI: 1.459–6.570), gender (IRR = 1.2, 95% CI: 1.0–5.1), awareness towards remote monitoring (IRR = 1.4, 95% CI: 1.1–2.7) were significantly associated with willingness to use information technologies.

**Conclusion:**

Improving the intention and skill of using computers should be a major point of attention for teaching hospitals who wish to improve their care providers' attitudes to remote monitoring and willingness in using information technologies. Besides, the awareness of professionals is crucial for improving willingness.

## Background

Diabetes mellitus (DM) is a group of chronic metabolic disorders that are associated with significant morbidity, mortality, and high health care cost [1]. Over the past years DM is becoming a major public health problem, According to the International Diabetes Federation (IDF) report, more than 463 million people affected by diabetes in the world, 2019 [2]. This global trend is particularly evident in Africa, where more than 19 million people are affected by DM. In Ethiopia, the prevalence is the highest in Africa with more than 1.7 million people live with DM [1, 2]. Hence, there is an urgent need to reduce the rising morbidity and mortality from diabetes.

The traditional models of care for chronic or diabetes care in Ethiopia is discontinuous, Healthcare providers review patients during face-to-face consultations but do not have access to their clinical status in between consultations [3]. Patients may develop complications as a result of events that go undetected during this interval, resulting in missed opportunities for early intervention to prevent adverse outcomes. Besides this, studies show diabetes patient management practice in Ethiopia has yet to progress to the point where it can be considered as effective [4].

Digitizing health systems are considered as a potential to improve healthcare services or possibly as an alternative in some healthcare areas such as patient management [5]. Technologies inpatient management practice can be an asynchronous and synchronous mode for data transmission by using telephones, mobile devices, computers, and Internet-connected devices. Also, they use a glucometer, mobile applications, Blood pressure measurements, pedometer, and telephones for recording patient data at distance [6].

In the Ethiopian Health Sector Transformation Plan, the Government had positioned the use of health information technology as a key transformation enabler to improve access and quality of health care [7, 8]. So far, different telemedicine systems has been deployed as a pilot in not more than ten hospitals in the country, these were limited to providing consultation between healthcare professionals [9, 10]. In the pilot implementation phase, user resistance was reported to be the primary hindering factor to its successful adaptation [9].

A study conducted in Addis Ababa indicated that a patient-centred approach could improve diabetes patient care [11]. However, there has been lack of report on electronic technologies interventions to support or manage diabetes or chronic care. The use of different electronic technologies such as voice calls, email, text messages (Short message service, also known as "SMS"), and video conferencing has a great potential to improve the management of diabetic care [12, 13].

In other settings, the use of text messaging for counselling and follow-up of diabetes patients remotely includes improvement in patient's self-management practice, medication adherence, and also reduction of diabetes complications, and several physician's Visits. Some interventional studies that are conducted in Bangladesh [14], Egypt [13], and Senegal [15] on DM patients had already shown this improvement. Also, Phone-based (voice calls) interventions, seem to improve blood glucose control and patient self-efficacy when these interventions are compared with the usual care [16]. In the same way, the use of internet-based technologies for close management of diabetic patients has shown a positive impact in the reduction of blood glucose level, comorbidities, clinical visits, and adherence to therapeutic and hygiene-dietary measures when compared with the usual care [17].

The perceived benefit of applying information technologies carry a great promise for diabetes patient management. However, to obtain the benefits of this technology, it is important to address factors related to health care providers' acceptance. Known deterrents of care provider's willingness to use information technologies include awareness, perceived usefulness, perceived easiness, and availability of technical support, attitude, and computer literacy [18–21].

Therefore, we assessed survey data from two teaching hospitals in Ethiopia to describe healthcare providers' attitudes and willingness to use information technologies for managing diabetes patients remotely.

## Methods

### Study design and setting

The survey protocol was approved by the ethical review committee of the Institute of public health, University of Gondar, Ethiopia, (No. IPH/837/02/2020), and conducted following the guidelines of the Declaration of Helsinki.

An institution-based cross-sectional study design was used to assess the attitude and willingness of healthcare workers towards using information technology for monitoring diabetes patients from February to March 2020 in the Amhara region, Ethiopia. The Amhara region is located in the North-Western and North-Central parts of Ethiopia. It has 10 administrative zones, one special zone, 181 woredas, and 78 urban centers. According to the Amhara Region health office report in 2019, the region has two teaching Referral hospitals, namely the University of Gondar and Tiebe-Ghion specialized teaching referral hospitals. These specialized teaching referral hospitals were similar in terms of staff and the scope of the service they provide.

### Study tool

Data were collected by using a structured self-administered questionnaire designed for the study. The design and development of the survey instrument were guided by the literature review and the questionnaire was adapted from various survey tools that had previously pilot-tested [21–24]. It also touches a number of factors previously identified from the literature that impact the attitude and willingness of health professionals regarding telehealth [20, 25–27]. The questionnaire consists of five main parts. Part 1 includes socio-demographic and access to basic technologies’ information of respondents (ten items), part 2 is related to organizational information (three items), part 3 assessed behavioral factors (seven items), part 4 included factors related to information technology (seven items), the final section of the questionnaire consisted of three items for attitude and five items for willingness assessment.

The attitude of the respondents was assessed by using an item rated on a five-point Likert scale that ranged from "1 = strongly disagree" to "5 = strongly agree," then scores of Liker scale statement was dichotomized into two. In this study, a score of less than ≤ 3 is labelled as "unfavorable or negative" and greater than 3.0 is labelled as a "favorable or positive" attitude [22]. In addition, respondents' level of willingness was assessed by using five items to be answered in either "Yes" or "No". They were asked about their willingness to use five distinct information technologies for supporting or monitoring diabetes patients remotely, which could allow them to better manage/support their patients namely voice calls, text messages, e-mail, social media, and video conferencing. A "yes" response was taken to mean that the care provider was willing, while the "No" response was regarded as unwilling. The result was computed by adding their response to each technology. A score of "1" will be given for "Yes" and "0" for "No." One can score a minimum of 0 and a maximum of 5 in this section [23]. The questions regarding attitude and willingness were validated and used in different studies of e-health and telehealth projects [22, 23, 28].

Furthermore, Content and face validation was conducted on the initial version of the questionnaire [29, 30]. The opinions of two experts who have an experience in the field of e-health served as a basis for content validation. The experts expressed their views related to the importance and relativity of the content. Efforts were made to develop a questionnaire that was brief and simple. The investigator made adjustments and administered the questionnaire to a small group of 20 physicians and nurses who were working at Tikur-Anbesa specialized teaching hospital to assess each questions in terms of clarity. The proposed changes were included in the final questionnaire. STATA version 14.1 was used for the calculation of reliability coefficients. Internal consistency was measured by Cronbach's alpha coefficient, while reproducibility was evaluated using intraclass correlation for each item in the attitude and willingness scales, the intraclass correlation coefficient in all subscales was above 0.7. Calculation for Cronbach's alpha was set at 0.72 for attitude, and 0.80 for willingness section.

### Respondent selection and data collection

The source population of this study was all physicians and nurses in the specialized teaching referral hospitals in the Amhara region. The study population of this study was physicians and nurses who were working permanently in the specialized teaching referral hospitals. All physicians and nurses who had served at least six months before the study were included in the study. All physicians and nurses who were on annual leave, sick leave, who left for a long time education were excluded from the study.

The Sample size was determined based on the assumption of the single population proportion formula. Since there was no prior study undertaken on a similar study population, with an estimated precision of 5% and the 95% confidence interval and a non-respondent rate of 10%. Therefore, the final sample size was 423.

During data collection, 1029 physicians and nurses were working in those hospitals permanently. Among those physicians and nurses, 423 physicians and nurses were randomly selected and included in the study. The sample was allocated to each hospital proportional to the number of physicians and nurses in each hospital. Then the sample was allocated to each department in the two hospitals proportional to the number of physicians and nurses in the department, and finally a simple random sampling technique was used to select the study participants from each department.

Data were collected using a structured self-administered questionnaire. The questionnaire was in prepared English and comprised of 36 questions. The same questionnaire was used for nurses and physicians. A paper-based version of the questionnaire was used to collect the data. The data collection process was conducted by using four data collectors and two supervisors, after one-day training. After the data collection, data were entered properly into Epi-data version 4.6 and exported to STATA 14.1 for analysis. In addition to prevent data loss electronic copies of data were stored and also shared with Health informatics department, university of Gondar and advisors.

### Statistical data analysis

The data were checked, cleaned, edited, and analyzed by using STATA version 14.1. Descriptive statistics (mean and percentage) were used to describe demographic characteristics and level of attitude and willingness. The binary logistic regression method was used to identify variables associated with the attitude of respondents. Also, the Poisson regression model was used to identify variables associated with the willingness of respondents. However, the assumption of Poisson regression was failed due to the existence of overdispersion on willingness data (variance of willingness data is greater than the mean), then negative binomial regression was fitted.

Furthermore, the results of logistic regression analysis were expressed as Odd ratios (OR) and results of negative binomial regression analysis were expressed as Incidence Rate Ratio (IRR), accompanied by 95% CI (confidence interval), and a p-value < 0.05 was calculated to evaluate statistical significance.

## Result

### Socio-demographic characteristics

Out of a total of 423 respondents, 406 completed the survey (response rate = 95.9%). The share of collected questionnaires in relation to dispatched questionnaires doesn't vary among the hospitals. The background information of the respondents can be seen in Table 1. A slim majority (57.6%) of the study respondents were below the age of 30 years, 61.3% were males. This was expected as more than 78% of physicians and nurses working in the Ethiopia healthcare are Males [31]. Out of respondents, (69.7%) were nurses, and 30.3% were physicians by profession. These results match numbers from Ethiopian national health workforce statistics, which show a large difference between the number of physicians and nurses in Ethiopia [31, 32].

**Table 1:** Socio-demographic characteristics stratified by the profession of participants at teaching hospitals in the Amhara region, 2020

Variable	Physician n (%)	Nurse's n (%)
Gender		
Male	90 (36.1)	159 (63.9)
Female	33 (21)	124 (79)
Age		
< 30	68 (29.1)	166 (70.9)
≥ 30	55 (32)	117 (68)
Educational level		
Medical doctor +	40 (100)	–
Medical degree	82 (100)	–
Master's degree	–	15 (100)
Bachelor	–	233 (100)
Diploma	–	36 (100)
Work experience		
0–5	89 (36.9)	152 (63.1)
6–10	17 (14.5)	100 (85.5)
> 10	17 (35.4)	31 (64.6)

In terms of educational level, the majority of respondents were bachelor (57.4%), and medical degree (20.2%) holders with a total contribution of (77.6%) respondents, as expected, considering the number of healthcare workers in Ethiopia. Most of the respondents, (59.4%) have working experience between 0 and 5 years and only (11.8%) have a working experience above 10 years.

### Access to basic technologies and pattern of usage

Table 2 shows that 95.1% of physicians and 53.4% of nurses own a personal computer. However, only 46.2% of the 95.1% physicians and 47% from 53.4% of nurses indicated their personal computer had internet capabilities. Regarding smartphones, more than 95.1% of physicians and 73.5% of nurses own smartphones. Furthermore, from the findings, barely 16% of the total respondents indicated they did not have a social media account.

**Table 2:** Access to basic technologies and patterns of usage among physicians and nurses at the University of Gondar and Tibebe Ghion teaching hospitals, 2020

Variables	Levels	Physician n (%)	Nurse n (%)
Having a personal computer	Yes	117 (95.1)	151 (53.4)
	No	6 (4.9)	132 (46.6)
Having an internet connection on a computer	Yes	54 (46.2)	71 (47)
	No	63 (53.8)	80 (53)
Having a smartphone	Yes	117 (95.1)	208 (73.5)
	No	6 (4.9)	75 (26.5)
Having an internet connection on a smartphone	Yes	116 (99.1)	191 (91.4)
	No	1 (0.9)	18 (8.6)
How often do you use a computer at work?	several times a day or daily	56 (45.5)	104 (36.7)
	weekly or rarely	58 (47.2)	140 (49.5)
	Never	9 (7.3)	39 (13.8)
How often do you use a computer at home?	several times a day or daily	88 (71.5)	96 (33.9)
	weekly or rarely	31 (25.2)	121 (42.8)
	Never	4 (3.3)	66 (23.3)
How often do you search for health-related information online?	several times a day or daily	91 (74)	101 (35.7)
	weekly or rarely	31 (25.2)	153 (54.1)
	Never	1 (0.8)	29 (10.2)
How often do you use e-mail to communicate with healthcare providers?	several times a day or daily	22 (17.9)	51 (18)
	weekly or rarely	89 (72.4)	174 (61.5)
	Never	12 (9.8)	58 (20.5)
How often do you download/ upload information through the internet?	several times a day or daily	67 (54.4)	97 (34.3)
	weekly or rarely	51 (41.5)	163 (57.6)
	Never	5 (4.1)	23 (8.1)

According to Table 2, in terms of home computer use, about 71.5% of physicians were daily computer users and only 7.3% of them never used computers at home. But, the computer use reported by nurses showed that not more than 37% of them used computers daily at home. In addition, physicians extensively search health-related information daily 74% than nurses 35.7%. Furthermore, 54.4% of physicians and 34.3% of nurses download or upload information using the internet daily. On the other hand, email seems to be much less utilized for communication among respondents.

### Respondents attitude towards remote monitoring and ICT tools

**Fig. 1:**
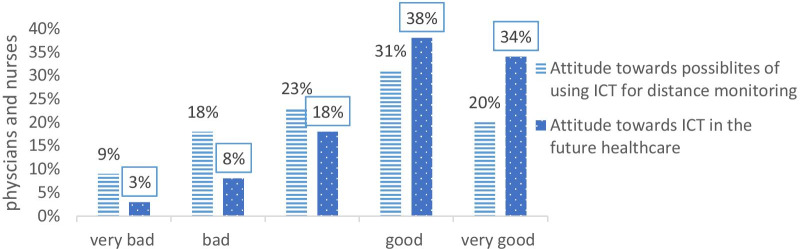
Attitudes of respondents towards current and future ICT tools in healthcare

**Fig. 2:**
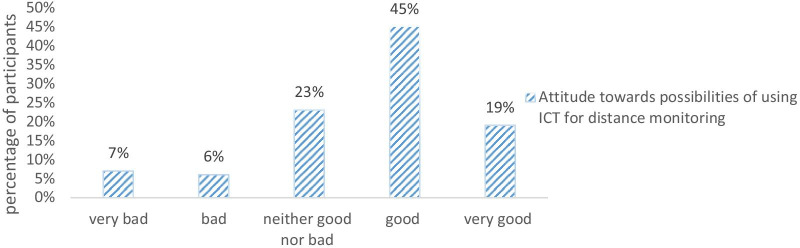
Attitudes of respondents towards remote monitoring through ICT tools

Figure 1 shows the attitude of care providers towards healthcare ICT today and in the future. In both cases, a majority of the respondent had positive opinions, and very few had negative opinions. As Fig. 2 shows, a majority of the respondents believed that the possibilities of remote monitoring of patients through ICT are good or very good. One-fourth of the respondents were neutral, leaving very few find this to be a bad or very bad method.

### Respondents willingness to use information technologies for remote monitoring

Of the total participants, 83% of them were willing to use one or more information technologies to support chronic patients. For instance, 70.4% of them were willing to voice calls. However, when compared to other low level of willingness was observed in social media (57.4%).

**Fig. 3:**
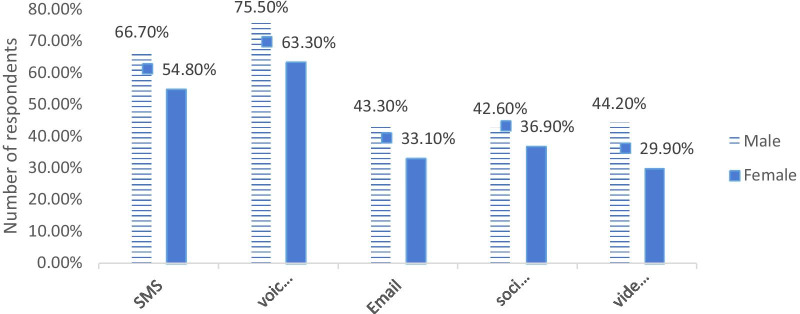
Willingness of respondents towards information technology tools by gender

**Fig. 4:**
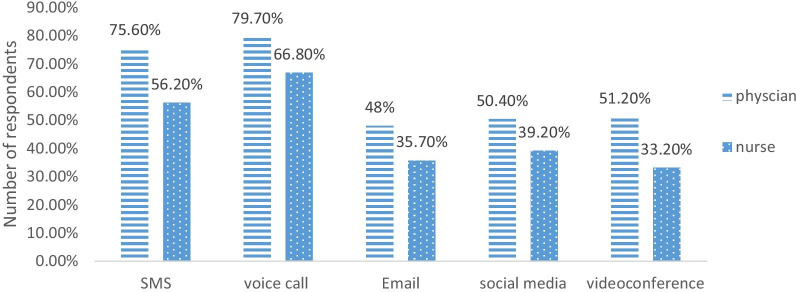
Willingness of respondents towards information technology tools by profession

As we see in Fig. 3, as for gender, 75% of males were willing to voice calls and 29% of females were willing in a videoconference. Refereeing to Fig. 4, as for profession, a slim majority of physicians were willing in social media and only 33.2% of nurses were willing towards videoconference.

### Factors associated with physicians and nurses attitude towards remote monitoring

In bivariate analysis, variables like access to a personal computer, Computer-related training, profession, participant innovativeness, and practice of communicating with patients through electronic technology tools were positively associated with the attitude of physicians and nurses towards remote monitoring. Table 3 shows the factor associated with respondent attitude toward remote monitoring. In multivariate analysis, respondents who owned a personal computer were about 2.6 times more likely to have a favorable attitude about remote monitoring (AOR = 2.6, 95% CI = 1.3–3.8) as compared to those who did not own a personal computer. Innovative respondents were about 2.7 times more likely to have a favorable attitude (AOR = 2.7, 95% CI = 1.8–4.3) as compared to non-innovative respondents.

**Table 3:** Bivariate and multivariate logistic regression factors associated with the attitude of physicians and nurses towards remote monitoring at teaching hospitals, 2020

Variable	Category	Attitude		Crude OR(95%CI)	Adjusted AOR(95%CI)
		Positive n (%)	Negative n (%)		
Gender	Male	162 (62.5)	87 (59.2)	1.2 [1.0–5.1]*	1.8 [.9–3.4]
	Female	97 (37.5)	60 (40.8)	1	1
Work experience	< 5 years	105 (40.7)	40 (27.4)	2.8 [1.4–5.7]*	1.8 [.5–6.5]
	> 10 years	81 (31.4)	50 (34.2)	1	1
Search health information through the internet	Daily	128 (49.9)	64 (43.5)	1.7 [1.5–6.6]*	.9 [0.8–1.1]
	Weekly	117 (45.2)	67 (45.6)	.8 [.2–2.7]	
	Never	14 (5.4)	16 (10.9)	1	1
Having a computer	Yes	169 (65.3)	99 (67.3)	1.9 [1.6–3.4]*	2.3 [1.3–3.8]**
	No	90 (34.7)	48 (32.7)	1	1
Profession	Physician	91 (35.1)	32 (21.8)	1.9 [1.2–3.1]*	1.7 [0.8–3.7]
	Nurses	168 (64.9)	115 (78.2)	1	1
Experience in supporting using ICT tools	Yes	150 (57.9)	64 (43.5)	1.7 [1.2–2.7]	1.7 [1.1–2.6]**
	No	109 (42.1)	83 (56.5)	1	1
Innovativeness	Innovative	182 (70.3)	69 (46.9)	2.7 [1.8–4.1]*	2.8 [1.8–4.3]***
	Non-innovative	77 (29.7)	78 (53.1)	1	1
Computer training	Yes	166 (64.1)	93 (35.9)	1.2 [.7–1.9]*	1.6 [1.0–3.2]
	No	101 (68.7)	46 (31.3)	1	1

*P-value ≤ 0.05 for bivariable analysis

**P-value < 0.01

***P-value < 0.001 for multivariable analysis, 1 = reference category

Furthermore, respondents who had experience in supporting/communicating with patients using information technology tools were about 1.7 times more likely to have a favorable attitude (AOR = 1.7, 95% CI = 1.1–2.6) as compared to respondents who had no experience to support/communicate with patients through information technology tools.

### Factors associated with physicians and nurses willingness to use information technology

The multivariable negative binomial regression model identified attitude (positive; 2.3, (95% CI: 1.1 to 4.7)), computer use (daily; 1.3, (95% CI: 1.2 to 3.0)), searching health information (daily; 1.7, (95% CI: 1.5 to 6.6)), awareness in telemonitoring (heard; 1.4, (95% CI: 1.1 to 2.7)), gender (Male; 1.2, (95% CI: 1.0 to 5.1)), as factors significantly associated with willingness to use different information technologies to support patients remotely (Table 4). Respondents with a favorable attitude towards remote monitoring of patients were 2.3 times more likely to be willing to use information technologies compared to the willingness of respondents with an unfavorable attitude towards remote monitoring.

**Table 4:** Result of negative binomial regression factors associated with willingness to use information technologies for chronic/diabetes patient management at teaching hospitals, 2020

Willingness in information technologies				
Variables	Category	coefficient	IRR	95% CI
Attitude towards remote monitoring	Positive	.826	2.285	1.112–4.695**
	Negative	0	1	
Use computer home	Daily	.2353	1.335	
	Weekly	−.049	0.952	0.814–1.14
	Never	0	1	
Search health information through the internet	Daily	.551	1.736	1.459–6.570**
	Weekly	−.220	.803	0.235–2.742
	Never	0	1	
	Heard	.346	1.414	1.084–2.663**
	Not heard	0	1	
Gender	Male	0.196	1.217	1.012–5.118***
	Female	0	1	

*TM* tele-monitoring

*P-value < 0.05

**P-value < 0.01

***P-value < 0.001, 1 = reference

Similarly, the willingness of respondents increased by 33.5% among those who used computers daily compared to the willingness of respondents who never used computers.

For searching for health-related information categories, the willingness of respondents increased by 73.6% among respondents who search for health-related information daily compared to the willingness of respondents who never search for health-related information. In addition, awareness of respondents towards telemonitoring has shown a significant impact on willingness, willingness to use information technologies inpatient management increased by 41.4% among respondents who have heard about telemonitoring compared to the willingness of respondents who did not hear. Furthermore, there was a 21.7% increase in willingness to use information technologies among male respondents compared to female respondents.

## Discussion

This paper attempt describes common information technologies used in the management of diabetes patients, and assess the attitude and willingness of physicians and nurses regarding electronic technologies for remote monitoring of diabetes patients.

The primary objective of this paper was to describe the attitude of care providers towards remote monitoring. To fulfill this overarching goal, the analysis focused on two parts. First, our descriptive analysis revealed that (64%) of respondents have a favorable attitude about using ICT tools for remote monitoring, which is by far less than the number of health professionals from other studies, which is (80%) [22].

Remote monitoring is a recent conception and requires gadgets such as smartphones and computers, in this study a majority of participants owned smartphones (80%) and.

personal computers (66%), which is higher than a previous study computer access were (36.7%) among health professionals in Ethiopia [27]. This finding is important since the lack of access to computers and smartphones can act as a barrier to the implantation of ICT [24]. Moreover, our study revealed that 51% of participants have a good attitude towards currently available ICT tools in healthcare. This finding is lower than other research studies conducted [22]. But 74% of them have a good opinion towards the future of ICT tools in health care.

Second, the result from our logistic regression indicated that computer-related training, technical skill, experience in supporting patients using ICT tools, and work experience had a significant association with having a positive attitude about remote monitoring of patients (*p* value < 0.05). Respondents who had good technical skills had a favorable attitude about using ICT for remote monitoring (AOR = 2.8, (95% CI):( 1.1, 7.1), implying that respondents' technical skill could have a positive correlation with their internet access, usages, and availability of infrastructure. This finding is in line with other research studies which indicate ICT skill could have a positive correlation with having a positive attitude.

Among organizational factors fitted into the logistic regression model, only computer-related training was found to affect attitude. Respondents who have previous computer-related training were more aware of TM (*p*-value = 0.021). The possible reason for this could be computer-related training was more likely to increase respondents' familiarity in using technologies [33].

To address the second objective, we assessed physicians' and nurses' self-reported interest in adopting information technologies, such as text messaging, voice calls, email, social media, and video conferencing, focusing on follow-up or monitoring of chronic patients remotely. Our research study indicated over 83% of respondents were willing to use one or more information technologies to support diabetes/chronic care management, which implies that these technologies permit further consideration for their potential to improve patient management practice in chronic care. Conversely, the willingness of participants in using email, videoconference, and social media were slightly lower compared to others, our findings suggest that alternative strategies like improving technical skill through training will be more productive in using this technology.

In addition, among all possible factors fitted into the negative binomial regression model, attitude, computer use, searching for health information, awareness, and gender were found to affect willingness to use information technologies. For instance, respondents who had a favorable attitude towards remote monitoring were more willing to information technologies. This result is in line with a study conducted in Canada [34]. Similarly, technical skill is postulated to be associated with the rising willingness to use information technologies with good technical skills are more receptive to use technologies [35].

We live in the era of exponential increase in both burden of diseases and health care needs of populations, this carries great burden for health systems management, especially in developing world. This increases the need on using innovative electronic technologies in order to address such needs within limited resources. In the context of Ethiopian healthcare, the use of electronic technologies has given a minimal attention by policymakers. However, recent years have brought a renewed interest and belief in its potential to transform healthcare quality and access [7, 8]. It is in this context that the idea of this paper was developed to assess whether the potential users are receptive to this technology, which could be very informative for initiating healthcare workers and policymakers in using this patient management option.

This research study has a few limitations. The study was conducted using only a quantitative approach. Future research studies should consider adding a qualitative approach to have more strength in findings. In addition, the study was conducted only in teaching hospitals, which may affect the generalizability of the findings to other settings. Future works would be better to incorporate settings other than teaching hospitals.

## Conclusion

Overall, this finding revealed that the majority of care providers' attitude towards remote monitoring was found to be good, and their willingness to use electronic technologies to facilitate diabetes patient management is high. Furthermore, in this thesis majority of respondents own a smartphone, and also they show significant willingness to support diabetes patients using mobile features like (text messaging, voice calls). Such an enabling finding should encourage policymakers, especially the Ministry of health, to first enhance, and then scale up the implementation and use of electronic technologies in care delivery. As a practical recommendation, the authors believe that supporting healthcare providers to use Mobile-based features for patient management would be feasible. This would ensure continuity of the treatment and disease management for common chronic conditions.

In regression analysis, having personal computers, self-perceived innovativeness, and previous experience of supporting patients using ICT tools were the most determinant factors for a favourable attitude of healthcare professionals in using information technologies to monitor patients remotely. The willingness of respondents was associated with having awareness in telemonitoring, attitude towards remote monitoring, gender, and technical skills. In addition to improving the intent and skill of using computers, system implementers need prior discussion and promotion of information technologies to improve healthcare providers' attitudes and willingness.

## Supplementary information

**Additional file 1**. Survey Questionnaire

## Data Availability

The datasets used and analyzed during the current study are available from the corresponding author on reasonable request.

## Abbreviations

AOR: Adjusted odds ratio
CI: Confidence Interval
DM: Diabetes Mellitus
ICT: Information Communication Technology
IDF: International Diabetes Federation
IRR: Incidence Rate Ratio
OR: Odds ratio
SMS: Short message service
