# Supplementary material for: Factors influencing healthcare providers’ attitude and willingness to use information technology in diabetes management
Source: BMC Med Inform Decis Mak. 2021 Jan 21;21:24. doi: 10.1186/s12911-021-01398-w (PMC7818744; doi:10.1186/s12911-021-01398-w)
Supplement: Supplementary file 1 — Additional file 1. Survey Questionnaire [file 12911_2021_1398_MOESM1_ESM.docx]

**Title page**

**Factors influencing healthcare providers’ attitude and willingness to use information technology in diabetes management.**

Binyam Tariku Seboka^1*^, Tesfahun Melese Yilma^2^ and Abraham Yeneneh Birhanu^3^.

^1^School of public health, Dilla University, Dilla, Ethiopia.

^2^Institute of public health, University of Gondar, Gondar, Ethiopia.

^3^Institute of public health, University of Gondar, Gondar, Ethiopia.

^*^Correspondence to Binyam Tariku Seboka [bini555tar@gmail.com]

**Email addresses:**

BT:bini555tar@gmail.com

TM:tesfahun.melese@uog.edu.et

AY:abreshyene@gmail.com

## **Survey Questionnaire**

Institution: _______________________ Questionnaire ID: ___________

Dear Hospital staff

My name is Binyam Tariku. I am conducting a research regarding the use of information technologies for Tele-monitoring/remote monitoring of diabetes patients at the University of Gondar and Tiebe ghion specialized teaching hospitals. Tele-monitoring(TM) or remote monitoring is the process of using information communication technology (ICT) to monitor/support the health status of a patient from a distance. In this Questionnaire, Tele-monitoring or remote monitoring refers to remote internet or Telephone based monitoring of blood glucose, blood pressure, and other signs and symptoms of diabetes patients. The recording devices are used by the patients in their own home environment and the generated data are transferred to health care providers over the internet, Telephone or mobile phone**.**

By answering all parts in this questionnaire you will help us to reach a fine result in the survey and your participation will be valued for future improvement of patient management practice. We confirm that the obtained information will be confidential.

Therefore I politely request your cooperation to respond at all or to withdraw in the meantime, but your input has great value for the success of my objective.

**Certificate of consent**

I understand that the findings of this research will be disseminated to Hospital management and decision-makers that will be useful as an input for intervention design

I voluntarily consent to participate in this study.

I Agree  I Disagree

If you have any enquiry regarding the study, please do not hesitate to contact on the following address. Phone No: **0945520778** or Email**:bini555tar@gmail.com**

**Part 1: Demographic and access to basic technology information**

Information here is about you and your workplace please answer by marking a tick (🗸) at the appropriate box that best describes yourself.

| Se. No. | Questions | Response | Skip |
| --- | --- | --- | --- |
| 101 | Gender | Male Female |  |
| 102 | Age In years | ____years |  |
| 103 | Medical specialty | Physician  Nurses |  |
| 104 | Level of education | GP Resident Specialist Diploma Bachelor Degree  Master’s Degree  other, please specify_ _ |  |
| 105 | Working experience In years | ____years |  |
| 106 | Do you have your own Computer/laptop | Yes  No | **If your answer is No to Q106, please go to Q108** |
| 107 | **If your answer is Yes to Q106**,On your computer, do you have internet access | Yes  No |  |
| 108 | Do you have your Own smartphone(phone that can install apps,have GPS) | Yes  No | **If your answer is No to Q108, please go to Q110** |
| 109 | **If your answer is Yes to Q108**,on your smartphone, do you have internet access | Yes  No |  |
| 110 | Do you have an account on social media? | Yes  No |  |

| **Part 2 : Organizational information**  Please indicate your opinion by ticking (🗸) at the appropriate box. | | | |
| --- | --- | --- | --- |
| 201 | Have you ever taken any computer-related training that can support your patient management? | Yes  No |  |
| 202 | Do you think that your organization has IT support staff? | Yes  No |  |
| 203 | Do you think you have internet access in your office at clinical practice? | Yes  No |  |

**Part 3: behavioral factors (Questions related to computer skill and innovation)**

For each of the questions below, please circle the number that best characterize your use.

| Se. No. | ***Please circle the number to which your use is exactly matching*** | Never | Rarely | Weekly | Daily | Several times a day |
| --- | --- | --- | --- | --- | --- | --- |
| 301 | How often do you use a computer at work? | 1 | 2 | 3 | 4 | 5 |
| 302 | How often do you use a computer at home? | 1 | 2 | 3 | 4 | 5 |
| 303 | How often do you search for health related information online? | 1 | 2 | 3 | 4 | 5 |
| 304 | How often do you use e-mail to Communicate with healthcare providers? | 1 | 2 | 3 | 4 | 5 |
| 305 | Have you been questioned by patients about online means of contacting you? | 1 | 2 | 3 | 4 | 5 |
| 306 | How often do you download/ upload information through internet? | 1 | 2 | 3 | 4 | 5 |

**Self-perceived innovativeness** *(Please encircle the number to show your answer)*

| Se. No. | Statement | Strongly Disagree | Disagree | Neither Disagree nor Agree | Agree | Strongly Agree |
| --- | --- | --- | --- | --- | --- | --- |
| 307 | If I heard about new information technology, I would look for ways to experiment with it | 1 | 2 | 3 | 4 | 5 |
| 308 | Have you ever heard about Tele-monitoring/remote-monitoring? | Yes  No | | | | |

**Part 4: Questions related information technology related factors**

Please read the following statements concerning the possible positive impacts that the use of information technologies may have in your practice and, circle the number that best characterize your perception ***,where 1 = Strongly Disagree, 2 = Disagree, 3 = Neither Disagree nor Agree, 4 = Agree, 5 = Strongly Agree***

| **Perceived usefulness***(Please encircle the number to show your answer )* | | | | | | |
| --- | --- | --- | --- | --- | --- | --- |
| Se. No. | Statement | Strongly Disagree Strongly Agree | | | | |
| 401 | I believe information technologies will positively affect the treatment plan. | 1 | 2 | 3 | 4 | 5 |
| 402 | I think it is possible to provide a more comprehensive diabetes care service. | 1 | 2 | 3 | 4 | 5 |
| 403 | It is efficient for diagnosing patients and scheduling. | 1 | 2 | 3 | 4 | 5 |
| 404 | I can precisely monitor the patient’s condition. | 1 | 2 | 3 | 4 | 5 |

- *Please read the following statements concerning easiness to use information technologies for patient management*

| **Perceived ease of use** *(Please encircle the number to show your answer )* | | | | | | |
| --- | --- | --- | --- | --- | --- | --- |
| Se. No. | Statement | Strongly Disagree Strongly Agree | | | | |
| 405 | It is easy to use the device for Tele-monitoring service. | 1 | 2 | 3 | 4 | 5 |
| 406 | It is easy to learn how to use the new device for Tele-monitoring. | 1 | 2 | 3 | 4 | 5 |
| 407 | It is easy to perform my job by using the Tele-monitoring service | 1 | 2 | 3 | 4 | 5 |

**Part 5: Attitudes towards ICT (information communication technology) tools**

| 501. What do you think about ICT as a tool in health care today? | 1 | 2 | 3 | 4 | 5 |
| --- | --- | --- | --- | --- | --- |
| 502. What do you think about the possibilities of ICT as a tool in health care in the future? | 1 | 2 | 3 | 4 | 5 |
| 503. What do you think of the possibility of remote monitoring / follow-up of patients via phone/Internet? | 1 | 2 | 3 | 4 | 5 |

**Where 1=Very bad 2=Bad 3=neither good nor bad 4= Good 5= Very good**

**Questions Related to willingness to use information technologies**

Please answer the following questions by putting a tick (🗸) at the appropriate box that show your willingness to include each modalities in patient management

| Se. No. | Questions | Response | Skip |
| --- | --- | --- | --- |
| 504 | Would you willing to use **SMS (text message)** for home follow-up/monitoring of diabetes/chronic patients? | Yes  No |  |
| 505 | Would you willing to use **Phone (voice call)** for home follow-up/monitoring of diabetes/chronic patients? | Yes  No |  |
| 506 | Would you willing to use **Email** for home follow-up/monitoring of diabetes/chronic patients? | Yes  No |  |
| 507 | Would you willing to use **Social media (online chatting)** for home follow-up/monitoring of diabetes/chronic patients? | Yes  No |  |
| 508 | Would you willing to use **Videoconferencing (video calls)** for home follow-up/monitoring of diabetes/chronic patients? | Yes  No |  |
